# The Micro-Organisms of Potable Water

**Published:** 1886-05

**Authors:** 


### The Micro-Organisms of Potable Water.

A writer in the *Revista Italiana di Igiene* has been studying the bacteria in the water supply of Monaco. He finds that the water on arriving in the city, whither it is conducted through iron pipes under a pressure of from five to six atmospheres, contain five micro-organisms per cubic centimeter. After standing twenty-four hours, the number of micro-organisms increases to twenty per cubic centimeter. In two days the number is 10,500, in four days 315,000, and on the fifth day there are *over half a million per cubic centimeter*. He has also ascertained that the development of the micro-organisms is not retarded, as is generally supposed, by the movement of the water. This he has demonstrated by placing the water in receptacles kept in constant motion. In mineral and carbonic acid water, bottled under pressure, however, the number of micros.opic organisms does not increase, but diminishes.

An autopsy was made by Drs. Cornil and Roux on the body of the Russian patient of Pasteur, who died of rabies in the Hotel Dieu. The bites on the face were very extensive in two places, the upper lip was pierced through and torn aside, leaving the gum exposed as far as the first molars. Another wound in the temporal region, just above the zygoma, was also very deep, and in it was found a fragment of a canine tooth of the wolf. The brain, medulla and spinal cord presented no abnormal appearance.—*Progrès Médical.*

---

We welcome the *Southern California Practitioner* in our list of exchanges. This is a new journal, published in Los Angeles, and devoted to general medicine, special attention being paid to the climatology of Southern California. The journal is neat in appearance, and ably edited. We have but one criticism to offer: the editors seem to have been imbued with an excessive amount of the anti-Chinese spirit.

---

At the annual election of the Buffalo Medical and Surgical Association, held April 6th, the following officers were elected: President, Dr. W. W. Potter; Vice-President, Dr. J. B. Coakley; Secretary, Dr. F. R. Campbell; Treasurer, Dr. F. L. Brecht; Librarian, Dr. P. W. Van Peyma.

---

The *Boston Medical and Surgical Journal* reports seven cases of poisoning by canned tomatoes. Dr. Doggett, who has been studying the subject, believes that there is an acid in tomatoes which attacks the tin or lead can, making corrosive salts.

---

Peyrot, of Paris, has made experiments in the transplantation of tendons. In a case where one of the popliteal tendons had been destroyed in a man, a fresh tendon was taken from a dog. This united and the function was preserved.

---

The Arch Duke, Charles Theodore of Bavaria, who is a thoroughly educated physician and a practitioner, has become a member of Pasteur's corps of assistants in Paris.

---

Atlanta, Georgia, is to have a polyclinic. The specialist is hungering and thirsting after fame and practice there as in other cities.

